# Bis­{(*E*)-3-[2-(hy­droxy­imino)­propan­amido]-2,2-dimethyl­propan-1-aminium} bis[μ-(*E*)-*N*-(3-amino-2,2-dimethyl­prop­yl)-2-(hy­droxy­imino)­propanamido­(2−)]bis­{[(*E*)-*N*-(3-amino-2,2-dimethyl­prop­yl)-2-(hy­droxy­imino)­propanamide]­copper(II)} bis­((*E*)-{3-[2-(hy­droxy­imino)­propanamido]-2,2-dimethyl­prop­yl}carbamate) acetonitrile disolvate

**DOI:** 10.1107/S160053681204620X

**Published:** 2012-11-14

**Authors:** Andrii I. Buvailo, Anna V. Pavlishchuk, Larysa V. Penkova, Natalia V. Kotova, Matti Haukka

**Affiliations:** a Department of Chemistry, Kiev National Taras Shevchenko University, Volodymyrska str. 62, Kiev 01601, Ukraine; b University of Joensuu, Department of Chemistry, PO Box 111, FI-80101 Joensuu, Finland

## Abstract

The reaction between copper(II) nitrate and (*E*)-*N*-(3-amino-2,2-dimethyl­prop­yl)-2-(hy­droxy­imino)­propanamide led to the formation of the dinuclear centrosymmetric copper(II) title complex, (C_8_H_18_N_3_O_2_)_2_[Cu_2_(C_8_H_15_N_3_O_2_)_2_(C_8_H_17_N_3_O_2_)_2_](C_9_H_16_N_3_O_4_)_2_·2CH_3_CN, in which an inversion center is located at the midpoint of the Cu_2_ unit in the center of the neutral [Cu_2_(C_8_H_15_N_3_O_2_)_2_(C_8_H_17_N_3_O_2_)_2_] complex fragment. The Cu^2+^ ions are connected by two N—O bridging groups [Cu⋯Cu separation = 4.0608 (5) Å] while the Cu^II^ ions are five-coordinated in a square-pyramidal N_4_O coordination environment. The complex mol­ecule co-crystallizes with two mol­ecules of acetonitrile, two mol­ecules of the protonated ligand (*E*)-3-[2-(hy­droxy­imino)­propanamido]-2,2-dimethyl­propan-1-aminium and two negatively charged (*E*)-{3-[2-(hy­droxy­imino)­propanamido]-2,2-dimethyl­prop­yl}carbamate anions, which were probably formed as a result of condensation between (*E*)-*N*-(3-amino-2,2-dimethyl­prop­yl)-2-(hy­droxy­imino)­propanamide and hydro­gencarbonate anions. In the crystal, the complex fragment [Cu_2_(C_8_H_15_N_3_O_2_)_2_(C_8_H_17_N_3_O_2_)_2_] and the ion pair C_8_H_18_N_3_O_2_^+.^C_9_H_16_N_3_O_4_^−^ are connected *via* an extended system of hydrogen bonds.

## Related literature

For properties of polynuclear complexes, see: Krämer & Fritsky (2000 ▶); Fritsky *et al.* (2001 ▶, 2003 ▶); Thompson (2002 ▶); Wörl *et al.* (2005 ▶); Bauer-Siebenlist *et al.* (2005 ▶); Thallapally *et al.* (2010 ▶); Cui *et al.* (2012 ▶); Beauvais *et al.* (2000 ▶). For studies of dinuclear copper(II) catecholase activity, see: Demmin *et al.* (1991 ▶); Monzani *et al.* (1998 ▶). For use of 2-hy­droxy­imino­propanoic acid derivatives as versatile ligands, see: Fritsky *et al.* (1998 ▶, 2006 ▶); Kanderal *et al.* (2005 ▶); Moroz *et al.* (2008 ▶, 2010 ▶, 2012 ▶); For the τ parameter, see: Addison *et al.* (1984 ▶). For related structures, see: Duda *et al.* (1997 ▶); Dobosz *et al.* (1999 ▶); Mokhir *et al.* (2002 ▶); Onindo *et al.* (1995 ▶); Petrusenko *et al.* (1997 ▶); Sliva *et al.* (1997 ▶); Dvorkin *et al.* (1990*a* ▶,*b* ▶); Lampeka *et al.* (1989 ▶); Skopenko *et al.* (1990 ▶). For carbon dioxide capture, see: Kovbasyuk *et al.* (1997 ▶); Pavlishchuk *et al.* (2002 ▶); Nanda *et al.* (2006 ▶).
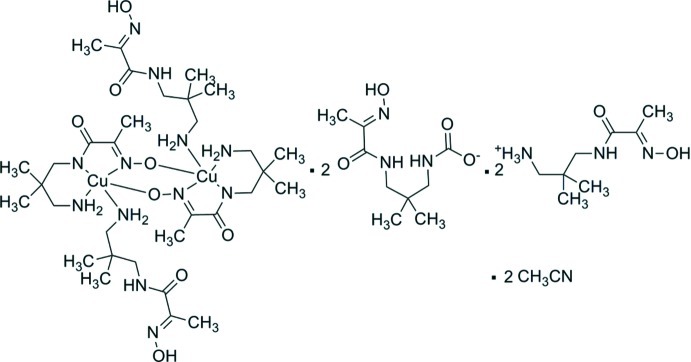


## Experimental

### 

#### Crystal data

(C_8_H_18_N_3_O_2_)_2_[Cu_2_(C_8_H_15_N_3_O_2_)_2_(C_8_H_17_N_3_O_2_)_2_] (C_9_H_16_N_3_O_4_)_2_·2C_2_H_3_N
*M* *_r_* = 1791.14
Triclinic, 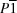

*a* = 9.3077 (3) Å
*b* = 12.9458 (6) Å
*c* = 19.8381 (6) Å
α = 107.875 (1)°
β = 98.461 (2)°
γ = 92.718 (2)°
*V* = 2239.34 (14) Å^3^
*Z* = 1
Mo *K*α radiation
μ = 0.55 mm^−1^
*T* = 120 K
0.17 × 0.14 × 0.11 mm

#### Data collection

Nonius KappaCCD diffractometer
Absorption correction: multi-scan (*SORTAV*; Blessing, 1995 ▶) *T*_min_ = 0.911, *T*_max_ = 0.944
32405 measured reflections
9811 independent reflections
7089 reflections with *I* > 2σ(*I*)
*R*_int_ = 0.074

#### Refinement

*R*[*F* ^2^ > 2σ(*F* ^2^)] = 0.053
*wR*(*F* ^2^) = 0.147
*S* = 1.05
9811 reflections
545 parameters
H-atom parameters constrained
Δρ_max_ = 0.78 e Å^−3^
Δρ_min_ = −0.70 e Å^−3^

### 

Data collection: *COLLECT* (Nonius, 2002 ▶); cell refinement: *DENZO*/*SCALEPACK* (Otwinowski & Minor, 1997 ▶); data reduction: *DENZO*/*SCALEPACK*; program(s) used to solve structure: *SHELXS97* (Sheldrick, 2008 ▶); program(s) used to refine structure: *SHELXL97* (Sheldrick, 2008 ▶); molecular graphics: *DIAMOND* (Brandenburg, 2009 ▶); software used to prepare material for publication: *SHELXL97*.

## Supplementary Material

Crystal structure: contains datablock(s) I, global. DOI: 10.1107/S160053681204620X/gg2096sup1.cif

Structure factors: contains datablock(s) I. DOI: 10.1107/S160053681204620X/gg2096Isup2.hkl

Additional supplementary materials:  crystallographic information; 3D view; checkCIF report

## Figures and Tables

**Table 1:** Selected bond lengths (Å)

Cu1—N1	1.984 (2)
Cu1—N2	1.957 (2)
Cu1—N3	2.000 (2)
Cu1—N4	2.041 (2)
Cu1—O1^i^	2.441 (2)

Symmetry code: (i) 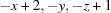.

**Table 2:** Hydrogen-bond geometry (Å, °)

*D*—H⋯*A*	*D*—H	H⋯*A*	*D*⋯*A*	*D*—H⋯*A*
O4—H4*O*⋯O2^i^	1.00	1.67	2.574 (3)	150
O6—H6*O*⋯O1^ii^	0.91	1.71	2.613 (3)	174
N3—H3*A*⋯O1^ii^	0.86	2.45	2.938 (3)	116
N3—H3*B*⋯O3	0.90	2.16	2.978 (3)	151
N4—H4*C*⋯O3	0.93	2.09	2.908 (3)	145
N4—H4*D*⋯O1	0.92	2.47	2.967 (3)	115
N4—H4*D*⋯N1^ii^	0.92	2.50	3.123 (3)	126
N7—H7*D*⋯O5^iii^	0.79	2.29	3.002 (3)	149
N7—H7*D*⋯O5	0.79	2.60	3.109 (3)	123
N7—H7*E*⋯O7^iii^	0.87	1.84	2.705 (3)	172
N7—H7*F*⋯O8^iv^	1.05	1.82	2.859 (3)	169
N7—H7*F*⋯O7^iv^	1.05	2.43	2.981 (3)	112
N10—H10*N*⋯N13^v^	0.98	2.27	3.107 (5)	143
N11—H11*N*⋯O7	0.90	1.95	2.773 (3)	151
O10—H10*O*⋯O8^vi^	0.86	1.77	2.626 (3)	169

Symmetry codes: (i) 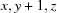; (ii) 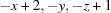; (iii) 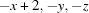; (iv) 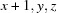; (v) 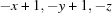; (vi) 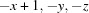.

**Fig. 1.**
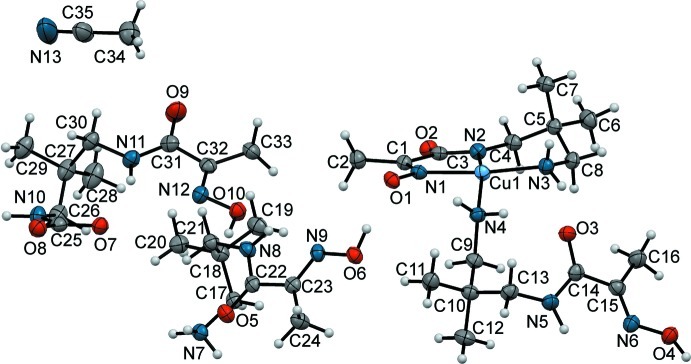
ORTEP view of (I) with the atomic numbering scheme (thermal ellipsoids are drawn at the 50% probability level).

**Fig. 2.**
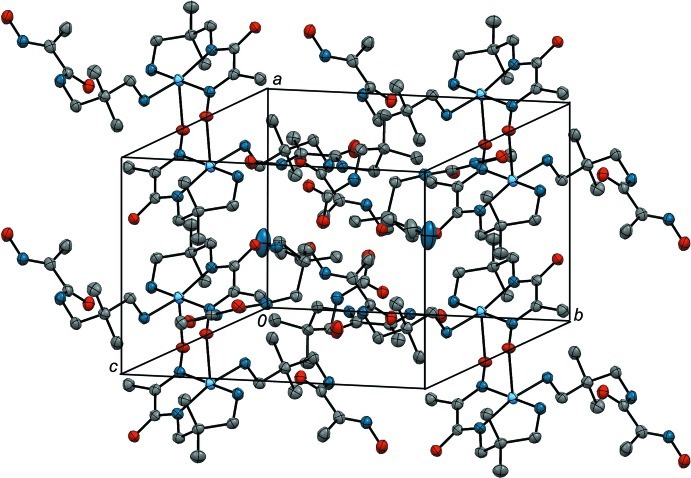
A view of the complete unit cell of the crystal structure of (I). Hydrogen atoms are omitted for clarity.

## supplementary crystallographic information

### Comment

Synthesis of the polydentate ligands is of particular interest due to their ability to form polynuclear complexes with different metal ions, which could be used in molecular magnetism (Thompson, 2002; Wörl *et al.*, 2005; Moroz *et al.*, 2010), bioinorganic modeling (Bauer-Siebenlist *et al.*, 2005), catalysis (Krämer *et al.*, 2000; Fritsky *et al.*, 2001; Fritsky *et al.*, 2003; Thallapally *et al.*, 2010), luminescence materials (Cui *et al.*, 2012) or sensors creation (Beauvais *et al.*, 2000). Dinuclear copper(II) complexes have received a lot of attention as far as they are suitable models for catecholase oxidase activity (Monzani *et al.*, 1998). It was found out that the separation between copper(II) ions dramatically influence the catalytic activity of the complex (Demmin *et al.*, 1991). So far investigations connected with the mechanism of copper(II) catalyzed cathechol oxidation are scarce, synthesis and structure determination of dinuclear copper(II) complexes with different Cu(II)—Cu(II) separations is of interest.

Amide derivatives of 2-hydroxyiminopropanoic acid have been widely used as versatile polynucleating ligands, in particular, for preparation of bi- and polynuclear complexes (Moroz *et al.*, 2008, 2010; 2012) and metal complexes with efficient stabilization of unusually high oxidation states of 3d-metal ions like copper(III) and nickel(III) (Fritsky *et al.*, 1998; Kanderal *et al.*, 2005; Fritsky *et al.*, 2006).

The title compound [Cu_2_(C_8_H_15_N_3_O_2_)_2_(C_8_H_17_N_3_O_2_)_2_]^.^2(C_9_H_16_N_3_O_4_)^-.^ 2(C_8_H_18_N_3_O_2_)^+.^ 2(CH_3_CN) (I) consist of a molecular complex fragment [Cu_2_(C_8_H_15_N_3_O_2_)_2_(C_8_H_17_N_3_O_2_)_2_], which is co-crystallized with two protonated ligands C_8_H_18_N_3_O_2_^+^, two deprotonated modified ligands C_9_H_16_N_3_O_4_^-^ and two acetonitrile molecules. The complex fragment [Cu_2_(C_8_H_15_N_3_O_2_)_2_(C_8_H_17_N_3_O_2_)_2_] contains two copper(II) ions, which are connected *via* two N—O bridging groups of the two doubly deprotonated ligands C_8_H_15_N_3_O_2_^2-^. The separation between copper (II) ions is 4.0608 (5) Å.

Each dianion C_8_H_15_N_3_O_2_^2-^ is coordinated to a copper(II) ion Cu1 *via* three nitrogen atoms N1, N2, N3 (from the oxime, amide and amino groups, respectively) and through oxygen atom O1 from the N—O group to a second copper(II) ion Cu1'. The coordination environment of each copper ion is completed by the nitrogen atom N4 from the monodentately coordinated neutral ligand molecule C_8_H_17_N_3_O_2_, thus resulting N_4_O donor set of copper(II) ions. The bond distances between copper(II) ion and nitrogen atoms N1 – N4 vary from the 1.957 (2) Å to 2.041 (2) Å, while the Cu1 – O1 bond is longer (2.4417 (1) Å), that is a consequence of the Jahn – Teller distortion. The copper(II) ions in (I) are located in the distorted square-pyramidal coordination environment, what confirms with low value of τ parameter (τ = 0.27) (Addison *et al.*, 1984). The C_8_H_15_N_3_O_22_^-^ dianions are coordinated to the copper(II) ions in a such way, that copper(II) and almost all non-hydrogen atoms of C_8_H_15_N_3_O_22_^-^ lie in one plane. The biggest deviation from the Cu1N1N2N3 plane among atoms of C_8_H_15_N_3_O_22_^-^ which are included in the chelate rings is observed for C5 (0.507 Å). Thereby, C_8_H_15_N_3_O_22_^-^ dianions accept an envelope conformation with a C5 atom being a flap atom. In the contrast to the C_8_H_15_N_3_O_22_^-^ dianions, both coordinated C_8_H_17_N_3_O_2_ ligand molecules and non-coordinated ion pair C_8_H_18_N_3_O_2_^+.^C_9_H_16_N_3_O_4_^-^ do not form a flat pseudo-macrocycle, which is observed in C_8_H_15_N_3_O_2_^2-^ dianions possibly owing to the coordination of copper(II) ions in the plane of this ligand.

In both coordinated and non-coordinated ligands the oxime group is situated in the *trans*-position with respect to the amide group and *anti*- with respect to the amide carbonyl which was early shown in the structures of similiar compounds - amide derivatives of 2-hydroxyiminopropanoic acid (Skopenko *et al.*, 1990; Onindo *et al.*, 1995; Duda *et al.*, 1997; Sliva *et al.*, 1997). The C=N and N—O bond lengths in the oxime moiety are typical for 2-hydroxyiminopropanoic acid and its amide derivatives (Lampeka *et al.*, 1989; Dvorkin *et al.*, 1990*a*, 1990*b*; Dobosz *et al.*, 1999; Mokhir *et al.*, 2002). The C—N and C—N bond lengths in the amine parts of the ligands are normal for aliphatic amines (Petrusenko *et al.*, 1997).

The non-coordinated anions C_9_H_16_N_3_O_4_^-^ in (I) possibly were formed due to the condensation processes between initial ligand C_8_H_17_N_3_O_2_ and carbon dioxide, which could been captured from air. Capture of CO_2_ from air and its following coordination or condensation with ligands in the complex composition is not rare (Kovbasyuk *et al.*, 1997; Pavlishchuk *et al.*, 2002; Nanda *et al.*, 2006). In the crystal packing of (I) complex fragment [Cu_2_(C_8_H_15_N_3_O_2_)_2_(C_8_H_17_N_3_O_2_)_2_] and the ion pair C_8_H_18_N_3_O_2_^+.^C_9_H_16_N_3_O_4_^-^ are connected *via* extended system of hydrogen bonds. Almost all H atoms in hydroxy, amino and imino groups in I are included in the formation of hydrogen bonding.

### Experimental

Synthesis of the ligand C_8_H_17_N_3_O_2_

A solution of the ethyl ester of 2-hydroxyiminopropanoic acid (13.1 g, 0.1 mol) in methanol (50 ml) was added to 1,3-diamino-2,2-dimethylpropane (5.1 g, 5.9 ml, 0.05 mol) in methanol (25 ml). The obtained mixture was stirred at 60°C for 30 min and after that kept at room temperature for 72 h. The solution was dried by rotary evaporation, yielding an oily residue. Its subsequent treatment with a small amount of water resulted in a white powder which was collected, washed with cold water and air-dried. The resulting product is fairly soluble in alcohols and DMSO and poorly soluble in hot water. Yield: 15.1 g (81%). Analysis calculated for C_8_H_17_N_3_O_2_: C 51.31, H 9.15, N 22.44%; found: C 51.72, H 9.19, N 22.37%.

Synthesis of [{Cu(C_8_H_15_N_3_O_2_)(C_8_H_17_N_3_O_2_)}_2_]&bull;[(C_8_H_18_N_3_O_2_)^+^(C_9_H_15_N_3_O_4_)^-^&shy;]_2_&bull;(CH_3_CN)_2_ (I)

A solution of copper (II) nitrate Cu(NO_3_)_2_^.^3H_2_O (0.242 g, 1 mmol) and C_8_H_17_N_3_O_2_ (0.561 g, 3 mmol) in methanol (10 ml) was stirred at 70°C for 30 min. To this 0.1*M* solution, NH_4_OH (3 ml) was added dropwise and the resulting mixture was stirred at 60°C during 30 min. Violet needle-like crystals suitable for X-ray analysis were obtained by salting out from the final solution with acetonitrile in a thin glass tube. Crystals were filtered out and then washed with acetonitrile and diethyl ester (yield 38%). Analysis calculated for C_70_H_136_Cu_2_N_26_O_20_: C 46.99, H 7.66, N 20.35%; found: C 45.11, H 8.13, N 19.42%.

### Refinement

The NH, NH_2_, NH_3_, and OH H atoms were located from the difference Fourier map but constrained to ride on their parent atom (*U*_iso_ = 1.5 (parent atom)). Other H atoms were positioned geometrically and allowed to ride on their parent atoms, with C—H = 0.98–0.99 Å, and *U*_iso_ = 1.2–1.5 *U*_eq_ (parent atom).

### Crystal data

**Table:**

(C_8_H_18_N_3_O_2_)_2_[Cu_2_(C_8_H_15_N_3_O_2_)_2_(C_8_H_17_N_3_O_2_)_2_] (C_9_H_16_N_3_O_4_)_2_·2C_2_H_3_N	*Z* = 1
*M**_r_* = 1791.14	*F*(000) = 958
Triclinic, *P*1	*D*_x_ = 1.328 Mg m^−^^3^
Hall symbol: -P 1	Mo *K*α radiation, λ = 0.71073 Å
*a* = 9.3077 (3) Å	Cell parameters from 23086 reflections
*b* = 12.9458 (6) Å	θ = 2.9–27.1°
*c* = 19.8381 (6) Å	µ = 0.55 mm^−^^1^
α = 107.875 (1)°	*T* = 120 K
β = 98.461 (2)°	Block, purple
γ = 92.718 (2)°	0.17 × 0.14 × 0.11 mm
*V* = 2239.34 (14) Å^3^	

### Data collection

**Table:**

Nonius KappaCCD diffractometer	9811 independent reflections
Radiation source: fine-focus sealed tube	7089 reflections with *I* > 2σ(*I*)
Horizontally mounted graphite crystal monochromator	*R*_int_ = 0.074
Detector resolution: 9 pixels mm^-1^	θ_max_ = 27.1°, θ_min_ = 3.9°
φ scans and ω scans with κ offset	*h* = −11→11
Absorption correction: multi-scan (*SORTAV*; Blessing, 1995)	*k* = −15→16
*T*_min_ = 0.911, *T*_max_ = 0.944	*l* = −25→25
32405 measured reflections	

### Refinement

**Table:**

Refinement on *F*^2^	Primary atom site location: structure-invariant direct methods
Least-squares matrix: full	Secondary atom site location: difference Fourier map
*R*[*F*^2^ > 2σ(*F*^2^)] = 0.053	Hydrogen site location: inferred from neighbouring sites
*wR*(*F*^2^) = 0.147	H-atom parameters constrained
*S* = 1.05	*w* = 1/[σ^2^(*F*_o_^2^) + (0.0692*P*)^2^ + 1.4972*P*] where *P* = (*F*_o_^2^ + 2*F*_c_^2^)/3
9811 reflections	(Δ/σ)_max_ = 0.001
545 parameters	Δρ_max_ = 0.78 e Å^−^^3^
0 restraints	Δρ_min_ = −0.70 e Å^−^^3^

### Special details

**Table:**

Geometry. All e.s.d.'s (except the e.s.d. in the dihedral angle between two l.s. planes) are estimated using the full covariance matrix. The cell e.s.d.'s are taken into account individually in the estimation of e.s.d.'s in distances, angles and torsion angles; correlations between e.s.d.'s in cell parameters are only used when they are defined by crystal symmetry. An approximate (isotropic) treatment of cell e.s.d.'s is used for estimating e.s.d.'s involving l.s. planes.
Refinement. Refinement of *F*^2^ against ALL reflections. The weighted *R*-factor *wR* and goodness of fit *S* are based on *F*^2^, conventional *R*-factors *R* are based on *F*, with *F* set to zero for negative *F*^2^. The threshold expression of *F*^2^ > σ(*F*^2^) is used only for calculating *R*-factors(gt) *etc*. and is not relevant to the choice of reflections for refinement. *R*-factors based on *F*^2^ are statistically about twice as large as those based on *F*, and *R*- factors based on ALL data will be even larger.

### Fractional atomic coordinates and isotropic or equivalent isotropic displacement parameters (Å^2^)

**Table:**

	*x*	*y*	*z*	*U*_iso_*/*U*_eq_	
Cu1	0.79591 (4)	0.03835 (3)	0.471381 (19)	0.02474 (11)	
O1	0.9971 (2)	0.00520 (16)	0.59142 (11)	0.0270 (4)	
O2	0.5459 (2)	−0.20172 (15)	0.49801 (11)	0.0282 (4)	
O3	0.8343 (2)	0.32441 (16)	0.44650 (11)	0.0316 (5)	
O4	0.5320 (2)	0.59018 (16)	0.43927 (12)	0.0341 (5)	
H4O	0.5444	0.6697	0.4459	0.051*	
O5	1.0148 (2)	−0.06821 (17)	0.05826 (11)	0.0313 (5)	
O6	1.0095 (2)	−0.12422 (16)	0.27643 (11)	0.0292 (4)	
H6O	1.0104	−0.0792	0.3216	0.044*	
O7	0.5645 (2)	0.13752 (16)	0.00244 (11)	0.0299 (5)	
O8	0.3750 (2)	0.14635 (17)	−0.07834 (12)	0.0346 (5)	
O9	0.8251 (3)	0.40092 (18)	0.23329 (14)	0.0489 (6)	
O10	0.7238 (2)	0.03350 (16)	0.18399 (12)	0.0324 (5)	
H10O	0.6854	−0.0205	0.1466	0.049*	
N1	0.8683 (3)	−0.02673 (18)	0.54686 (13)	0.0244 (5)	
N2	0.6419 (3)	−0.08185 (18)	0.44696 (13)	0.0255 (5)	
N3	0.7251 (3)	0.08880 (19)	0.38775 (13)	0.0282 (5)	
H3A	0.7873	0.0676	0.3598	0.042*	
H3B	0.7311	0.1605	0.3934	0.042*	
N4	0.9173 (3)	0.18149 (18)	0.53062 (13)	0.0256 (5)	
H4C	0.9301	0.2215	0.4996	0.038*	
H4D	1.0061	0.1569	0.5410	0.038*	
N5	0.8510 (3)	0.46726 (19)	0.54814 (14)	0.0296 (5)	
H5N	0.8229	0.5393	0.5632	0.044*	
N6	0.6524 (3)	0.55520 (19)	0.47341 (14)	0.0301 (6)	
N7	1.2786 (3)	0.03651 (19)	0.01429 (13)	0.0272 (5)	
H7D	1.1936	0.0243	−0.0002	0.041*	
H7E	1.3216	−0.0233	0.0095	0.041*	
H7F	1.3040	0.0833	−0.0181	0.041*	
N8	1.0700 (3)	0.07386 (19)	0.16118 (14)	0.0284 (5)	
H8N	1.0818	0.0910	0.2081	0.043*	
N9	1.0319 (3)	−0.05481 (19)	0.23722 (13)	0.0263 (5)	
N10	0.5814 (3)	0.2593 (2)	−0.05759 (15)	0.0345 (6)	
H10N	0.5339	0.2872	−0.0951	0.052*	
N11	0.7026 (3)	0.30587 (19)	0.12196 (14)	0.0301 (6)	
H11N	0.6605	0.2392	0.0949	0.045*	
N12	0.7126 (3)	0.12354 (19)	0.16016 (14)	0.0278 (5)	
N13	0.4204 (6)	0.6143 (3)	0.1678 (2)	0.0866 (15)	
C1	0.7829 (3)	−0.1065 (2)	0.54971 (15)	0.0255 (6)	
C2	0.8168 (4)	−0.1687 (2)	0.60094 (17)	0.0322 (7)	
H2A	0.9003	−0.2102	0.5894	0.048*	
H2B	0.7318	−0.2191	0.5972	0.048*	
H2C	0.8406	−0.1179	0.6501	0.048*	
C3	0.6456 (3)	−0.1336 (2)	0.49430 (15)	0.0241 (6)	
C4	0.5117 (3)	−0.1048 (2)	0.39132 (16)	0.0272 (6)	
H4A	0.4321	−0.0655	0.4125	0.033*	
H4B	0.4799	−0.1838	0.3755	0.033*	
C5	0.5350 (3)	−0.0719 (2)	0.32556 (16)	0.0262 (6)	
C6	0.3899 (4)	−0.0977 (3)	0.27351 (18)	0.0367 (7)	
H6A	0.3560	−0.1750	0.2616	0.055*	
H6B	0.4035	−0.0818	0.2295	0.055*	
H6C	0.3174	−0.0528	0.2960	0.055*	
C7	0.6526 (3)	−0.1355 (2)	0.28899 (17)	0.0300 (6)	
H7A	0.6203	−0.2137	0.2719	0.045*	
H7B	0.7437	−0.1218	0.3236	0.045*	
H7C	0.6690	−0.1116	0.2482	0.045*	
C8	0.5755 (3)	0.0517 (2)	0.34610 (16)	0.0278 (6)	
H8A	0.5670	0.0722	0.3016	0.033*	
H8B	0.5045	0.0904	0.3749	0.033*	
C9	0.8481 (3)	0.2553 (2)	0.58640 (16)	0.0266 (6)	
H9A	0.8221	0.2155	0.6190	0.032*	
H9B	0.7560	0.2737	0.5628	0.032*	
C10	0.9404 (3)	0.3622 (2)	0.63210 (16)	0.0279 (6)	
C11	1.0858 (4)	0.3386 (2)	0.66935 (18)	0.0334 (7)	
H11A	1.1402	0.4074	0.7007	0.050*	
H11B	1.1433	0.3018	0.6330	0.050*	
H11C	1.0668	0.2916	0.6981	0.050*	
C12	0.8516 (4)	0.4234 (3)	0.68900 (18)	0.0369 (7)	
H12A	0.8324	0.3785	0.7191	0.055*	
H12B	0.7587	0.4382	0.6650	0.055*	
H12C	0.9071	0.4924	0.7191	0.055*	
C13	0.9769 (3)	0.4349 (2)	0.58719 (17)	0.0296 (6)	
H13A	1.0370	0.5016	0.6196	0.036*	
H13B	1.0370	0.3955	0.5521	0.036*	
C14	0.7896 (3)	0.4098 (2)	0.48086 (17)	0.0279 (6)	
C15	0.6606 (3)	0.4527 (2)	0.44730 (16)	0.0294 (6)	
C16	0.5574 (4)	0.3726 (3)	0.38713 (19)	0.0385 (8)	
H16A	0.4661	0.4049	0.3786	0.058*	
H16B	0.5371	0.3066	0.3998	0.058*	
H16C	0.6013	0.3537	0.3435	0.058*	
C17	1.3337 (3)	0.0845 (2)	0.09269 (16)	0.0264 (6)	
H17A	1.4401	0.1047	0.0999	0.032*	
H17B	1.3188	0.0280	0.1158	0.032*	
C18	1.2620 (3)	0.1849 (2)	0.13065 (16)	0.0285 (6)	
C19	1.3412 (4)	0.2267 (3)	0.20854 (18)	0.0379 (7)	
H19A	1.3309	0.1698	0.2311	0.057*	
H19B	1.2985	0.2915	0.2349	0.057*	
H19C	1.4449	0.2454	0.2095	0.057*	
C20	1.2765 (4)	0.2735 (2)	0.09419 (18)	0.0330 (7)	
H20A	1.2375	0.3396	0.1215	0.050*	
H20B	1.2216	0.2470	0.0451	0.050*	
H20C	1.3796	0.2902	0.0928	0.050*	
C21	1.0987 (3)	0.1567 (2)	0.12810 (16)	0.0279 (6)	
H21A	1.0561	0.2237	0.1529	0.033*	
H21B	1.0494	0.1307	0.0773	0.033*	
C22	1.0300 (3)	−0.0316 (2)	0.12427 (16)	0.0258 (6)	
C23	1.0066 (3)	−0.1032 (2)	0.16946 (16)	0.0256 (6)	
C24	0.9528 (4)	−0.2213 (2)	0.13348 (17)	0.0334 (7)	
H24A	1.0145	−0.2662	0.1549	0.050*	
H24B	0.9569	−0.2409	0.0820	0.050*	
H24C	0.8518	−0.2338	0.1400	0.050*	
C25	0.5051 (4)	0.1773 (2)	−0.04470 (17)	0.0299 (7)	
C26	0.7333 (4)	0.2957 (3)	−0.02801 (19)	0.0364 (7)	
H26A	0.7826	0.3057	−0.0668	0.044*	
H26B	0.7782	0.2370	−0.0126	0.044*	
C27	0.7646 (3)	0.4020 (3)	0.03606 (19)	0.0345 (7)	
C28	0.9282 (4)	0.4177 (3)	0.0648 (2)	0.0469 (9)	
H28A	0.9523	0.4873	0.1036	0.070*	
H28B	0.9821	0.4177	0.0259	0.070*	
H28C	0.9553	0.3579	0.0833	0.070*	
C29	0.7196 (4)	0.4993 (3)	0.0118 (2)	0.0422 (8)	
H29A	0.6176	0.4845	−0.0124	0.063*	
H29B	0.7821	0.5105	−0.0215	0.063*	
H29C	0.7301	0.5650	0.0538	0.063*	
C30	0.6790 (3)	0.3988 (2)	0.09562 (18)	0.0316 (7)	
H30A	0.7069	0.4671	0.1364	0.038*	
H30B	0.5736	0.3965	0.0774	0.038*	
C31	0.7718 (3)	0.3139 (2)	0.18701 (17)	0.0308 (7)	
C32	0.7833 (3)	0.2103 (2)	0.20590 (16)	0.0277 (6)	
C33	0.8753 (4)	0.2187 (3)	0.27622 (17)	0.0332 (7)	
H33A	0.8243	0.2552	0.3156	0.050*	
H33B	0.9686	0.2611	0.2813	0.050*	
H33C	0.8932	0.1455	0.2779	0.050*	
C35	0.4618 (5)	0.5755 (3)	0.2090 (2)	0.0498 (9)	
C34	0.5186 (4)	0.5279 (3)	0.2630 (2)	0.0509 (9)	
H34A	0.6131	0.5014	0.2539	0.076*	
H34B	0.4502	0.4669	0.2613	0.076*	
H34C	0.5313	0.5831	0.3106	0.076*	

### Atomic displacement parameters (Å^2^)

**Table:**

	*U*^11^	*U*^22^	*U*^33^	*U*^12^	*U*^13^	*U*^23^
Cu1	0.0265 (2)	0.02338 (18)	0.02322 (19)	−0.00097 (13)	0.00151 (14)	0.00753 (13)
O1	0.0222 (10)	0.0343 (10)	0.0229 (10)	0.0009 (8)	0.0021 (8)	0.0076 (8)
O2	0.0275 (11)	0.0248 (9)	0.0332 (12)	0.0000 (8)	0.0076 (9)	0.0096 (8)
O3	0.0405 (13)	0.0261 (10)	0.0273 (11)	0.0055 (9)	0.0080 (9)	0.0059 (9)
O4	0.0378 (13)	0.0279 (10)	0.0354 (12)	0.0057 (9)	0.0006 (10)	0.0107 (9)
O5	0.0335 (12)	0.0354 (11)	0.0243 (11)	−0.0005 (9)	0.0031 (9)	0.0098 (9)
O6	0.0332 (12)	0.0321 (10)	0.0245 (11)	0.0014 (9)	0.0074 (9)	0.0115 (9)
O7	0.0327 (12)	0.0276 (10)	0.0310 (12)	0.0034 (8)	0.0065 (9)	0.0112 (9)
O8	0.0354 (13)	0.0332 (11)	0.0326 (12)	0.0019 (9)	0.0009 (10)	0.0093 (9)
O9	0.0582 (17)	0.0293 (12)	0.0479 (15)	−0.0004 (11)	−0.0110 (12)	0.0058 (11)
O10	0.0368 (12)	0.0290 (10)	0.0321 (12)	−0.0003 (9)	0.0025 (10)	0.0125 (9)
N1	0.0246 (13)	0.0239 (11)	0.0220 (12)	0.0006 (9)	0.0030 (10)	0.0041 (9)
N2	0.0243 (13)	0.0256 (11)	0.0265 (13)	−0.0001 (9)	0.0043 (10)	0.0083 (10)
N3	0.0296 (14)	0.0273 (12)	0.0284 (13)	0.0021 (10)	0.0035 (11)	0.0109 (10)
N4	0.0271 (13)	0.0233 (11)	0.0260 (13)	0.0024 (9)	0.0052 (10)	0.0069 (10)
N5	0.0338 (14)	0.0238 (11)	0.0284 (14)	0.0028 (10)	0.0016 (11)	0.0058 (10)
N6	0.0309 (14)	0.0294 (13)	0.0329 (14)	0.0049 (10)	0.0062 (11)	0.0135 (11)
N7	0.0225 (12)	0.0307 (12)	0.0267 (13)	0.0024 (10)	0.0023 (10)	0.0074 (10)
N8	0.0324 (14)	0.0309 (12)	0.0231 (13)	0.0021 (10)	0.0083 (10)	0.0089 (10)
N9	0.0244 (13)	0.0316 (12)	0.0264 (13)	0.0025 (10)	0.0056 (10)	0.0137 (10)
N10	0.0347 (15)	0.0373 (14)	0.0362 (15)	0.0028 (11)	0.0061 (12)	0.0185 (12)
N11	0.0297 (14)	0.0254 (12)	0.0350 (15)	−0.0005 (10)	0.0038 (11)	0.0104 (11)
N12	0.0257 (13)	0.0279 (12)	0.0317 (14)	0.0023 (10)	0.0052 (11)	0.0118 (11)
N13	0.158 (5)	0.056 (2)	0.046 (2)	0.013 (3)	0.003 (3)	0.0227 (19)
C1	0.0282 (15)	0.0254 (13)	0.0238 (15)	0.0050 (11)	0.0069 (12)	0.0077 (11)
C2	0.0344 (17)	0.0332 (15)	0.0307 (17)	0.0011 (13)	0.0040 (13)	0.0138 (13)
C3	0.0258 (15)	0.0210 (12)	0.0237 (14)	0.0019 (11)	0.0073 (11)	0.0032 (11)
C4	0.0240 (15)	0.0279 (14)	0.0271 (15)	−0.0001 (11)	0.0033 (12)	0.0057 (12)
C5	0.0234 (15)	0.0288 (14)	0.0251 (15)	0.0037 (11)	0.0022 (12)	0.0074 (12)
C6	0.0302 (17)	0.0444 (18)	0.0318 (18)	0.0011 (13)	−0.0025 (13)	0.0106 (14)
C7	0.0311 (16)	0.0296 (14)	0.0271 (16)	0.0034 (12)	0.0067 (13)	0.0049 (12)
C8	0.0250 (15)	0.0310 (14)	0.0272 (16)	0.0050 (12)	0.0012 (12)	0.0101 (12)
C9	0.0270 (15)	0.0273 (14)	0.0247 (15)	0.0005 (11)	0.0060 (12)	0.0067 (12)
C10	0.0285 (16)	0.0262 (14)	0.0265 (15)	0.0006 (11)	0.0044 (12)	0.0051 (12)
C11	0.0329 (17)	0.0278 (14)	0.0341 (17)	−0.0018 (12)	−0.0048 (13)	0.0074 (13)
C12	0.0412 (19)	0.0358 (16)	0.0292 (17)	0.0032 (14)	0.0096 (14)	0.0021 (13)
C13	0.0304 (16)	0.0230 (13)	0.0330 (17)	−0.0002 (11)	0.0028 (13)	0.0069 (12)
C14	0.0314 (16)	0.0240 (13)	0.0310 (16)	0.0008 (11)	0.0075 (13)	0.0120 (12)
C15	0.0345 (17)	0.0279 (14)	0.0258 (16)	0.0003 (12)	0.0068 (13)	0.0082 (12)
C16	0.042 (2)	0.0308 (16)	0.0371 (19)	0.0056 (14)	−0.0031 (15)	0.0072 (14)
C17	0.0220 (14)	0.0324 (14)	0.0256 (15)	0.0024 (11)	0.0038 (11)	0.0105 (12)
C18	0.0265 (16)	0.0297 (14)	0.0289 (16)	0.0002 (12)	0.0063 (12)	0.0083 (12)
C19	0.0328 (18)	0.0425 (18)	0.0328 (18)	−0.0012 (14)	0.0020 (14)	0.0061 (14)
C20	0.0327 (17)	0.0296 (15)	0.0380 (18)	0.0005 (12)	0.0111 (14)	0.0107 (13)
C21	0.0292 (16)	0.0284 (14)	0.0273 (16)	0.0028 (12)	0.0073 (12)	0.0096 (12)
C22	0.0196 (14)	0.0328 (14)	0.0253 (15)	0.0039 (11)	0.0030 (11)	0.0097 (12)
C23	0.0184 (14)	0.0304 (14)	0.0275 (16)	0.0020 (11)	0.0033 (11)	0.0086 (12)
C24	0.0373 (18)	0.0308 (15)	0.0297 (17)	−0.0004 (13)	0.0080 (14)	0.0057 (13)
C25	0.0352 (18)	0.0266 (14)	0.0298 (16)	0.0080 (12)	0.0119 (14)	0.0079 (12)
C26	0.0319 (17)	0.0404 (17)	0.048 (2)	0.0085 (13)	0.0181 (15)	0.0248 (15)
C27	0.0249 (16)	0.0354 (16)	0.050 (2)	0.0013 (12)	0.0072 (14)	0.0231 (15)
C28	0.0253 (17)	0.053 (2)	0.071 (3)	0.0008 (15)	0.0069 (17)	0.034 (2)
C29	0.0353 (19)	0.0374 (17)	0.062 (2)	0.0030 (14)	0.0099 (17)	0.0261 (17)
C30	0.0291 (16)	0.0271 (14)	0.0401 (18)	0.0018 (12)	0.0044 (14)	0.0135 (13)
C31	0.0266 (16)	0.0303 (15)	0.0327 (17)	0.0020 (12)	0.0047 (13)	0.0061 (13)
C32	0.0247 (15)	0.0326 (15)	0.0259 (15)	0.0046 (12)	0.0075 (12)	0.0076 (12)
C33	0.0377 (18)	0.0357 (16)	0.0239 (16)	0.0035 (13)	0.0061 (13)	0.0060 (13)
C35	0.074 (3)	0.0369 (18)	0.037 (2)	0.0001 (18)	0.0112 (19)	0.0097 (16)
C34	0.046 (2)	0.059 (2)	0.052 (2)	0.0056 (18)	0.0101 (18)	0.0238 (19)

### Geometric parameters (Å, º)

**Table:**

Cu1—N1	1.984 (2)	C8—H8A	0.9900
Cu1—N2	1.957 (2)	C8—H8B	0.9900
Cu1—N3	2.000 (2)	C9—C10	1.537 (4)
Cu1—N4	2.041 (2)	C9—H9A	0.9900
Cu1—O1^i^	2.441 (2)	C9—H9B	0.9900
O1—N1	1.343 (3)	C10—C11	1.535 (4)
O2—C3	1.276 (3)	C10—C13	1.539 (4)
O3—C14	1.234 (3)	C10—C12	1.540 (4)
O4—N6	1.390 (3)	C11—H11A	0.9800
O4—H4O	0.9950	C11—H11B	0.9800
O5—C22	1.232 (4)	C11—H11C	0.9800
O6—N9	1.384 (3)	C12—H12A	0.9800
O6—H6O	0.9050	C12—H12B	0.9800
O7—C25	1.271 (4)	C12—H12C	0.9800
O8—C25	1.275 (4)	C13—H13A	0.9900
O9—C31	1.240 (4)	C13—H13B	0.9900
O10—N12	1.389 (3)	C14—C15	1.499 (4)
O10—H10O	0.8640	C15—C16	1.494 (4)
N1—C1	1.293 (4)	C16—H16A	0.9800
N2—C3	1.308 (4)	C16—H16B	0.9800
N2—C4	1.465 (4)	C16—H16C	0.9800
N3—C8	1.485 (4)	C17—C18	1.526 (4)
N3—H3A	0.8568	C17—H17A	0.9900
N3—H3B	0.8984	C17—H17B	0.9900
N4—C9	1.474 (4)	C18—C19	1.529 (4)
N4—H4C	0.9349	C18—C21	1.536 (4)
N4—H4D	0.9174	C18—C20	1.543 (4)
N5—C14	1.337 (4)	C19—H19A	0.9800
N5—C13	1.458 (4)	C19—H19B	0.9800
N5—H5N	0.9502	C19—H19C	0.9800
N6—C15	1.279 (4)	C20—H20A	0.9800
N7—C17	1.487 (4)	C20—H20B	0.9800
N7—H7D	0.7915	C20—H20C	0.9800
N7—H7E	0.8748	C21—H21A	0.9900
N7—H7F	1.0512	C21—H21B	0.9900
N8—C22	1.341 (4)	C22—C23	1.503 (4)
N8—C21	1.452 (4)	C23—C24	1.499 (4)
N8—H8N	0.8760	C24—H24A	0.9800
N9—C23	1.277 (4)	C24—H24B	0.9800
N10—C25	1.358 (4)	C24—H24C	0.9800
N10—C26	1.445 (4)	C26—C27	1.541 (5)
N10—H10N	0.9806	C26—H26A	0.9900
N11—C31	1.326 (4)	C26—H26B	0.9900
N11—C30	1.465 (4)	C27—C28	1.526 (5)
N11—H11N	0.8987	C27—C30	1.529 (4)
N12—C32	1.283 (4)	C27—C29	1.535 (4)
N13—C35	1.115 (5)	C28—H28A	0.9800
C1—C2	1.488 (4)	C28—H28B	0.9800
C1—C3	1.507 (4)	C28—H28C	0.9800
C2—H2A	0.9800	C29—H29A	0.9800
C2—H2B	0.9800	C29—H29B	0.9800
C2—H2C	0.9800	C29—H29C	0.9800
C4—C5	1.531 (4)	C30—H30A	0.9900
C4—H4A	0.9900	C30—H30B	0.9900
C4—H4B	0.9900	C31—C32	1.504 (4)
C5—C6	1.528 (4)	C32—C33	1.496 (4)
C5—C7	1.535 (4)	C33—H33A	0.9800
C5—C8	1.538 (4)	C33—H33B	0.9800
C6—H6A	0.9800	C33—H33C	0.9800
C6—H6B	0.9800	C35—C34	1.442 (6)
C6—H6C	0.9800	C34—H34A	0.9800
C7—H7A	0.9800	C34—H34B	0.9800
C7—H7B	0.9800	C34—H34C	0.9800
C7—H7C	0.9800		
			
N1—Cu1—N2	81.63 (10)	H12B—C12—H12C	109.5
N1—Cu1—N3	173.95 (10)	N5—C13—C10	115.2 (2)
N1—Cu1—N4	89.54 (10)	N5—C13—H13A	108.5
N2—Cu1—N3	95.35 (10)	C10—C13—H13A	108.5
N2—Cu1—N4	157.75 (10)	N5—C13—H13B	108.5
N3—Cu1—N4	95.10 (10)	C10—C13—H13B	108.5
N6—O4—H4O	112.4	H13A—C13—H13B	107.5
N9—O6—H6O	104.4	O3—C14—N5	122.8 (3)
N12—O10—H10O	103.6	O3—C14—C15	119.9 (3)
C1—N1—O1	120.8 (2)	N5—C14—C15	117.3 (2)
C1—N1—Cu1	114.9 (2)	N6—C15—C16	127.2 (3)
O1—N1—Cu1	124.36 (17)	N6—C15—C14	115.6 (3)
C3—N2—C4	117.5 (2)	C16—C15—C14	117.2 (3)
C3—N2—Cu1	114.07 (19)	C15—C16—H16A	109.5
C4—N2—Cu1	127.20 (18)	C15—C16—H16B	109.5
C8—N3—Cu1	120.40 (18)	H16A—C16—H16B	109.5
C8—N3—H3A	109.0	C15—C16—H16C	109.5
Cu1—N3—H3A	103.3	H16A—C16—H16C	109.5
C8—N3—H3B	101.7	H16B—C16—H16C	109.5
Cu1—N3—H3B	119.6	N7—C17—C18	114.9 (2)
H3A—N3—H3B	101.0	N7—C17—H17A	108.5
C9—N4—Cu1	115.68 (18)	C18—C17—H17A	108.5
C9—N4—H4C	103.7	N7—C17—H17B	108.5
Cu1—N4—H4C	107.6	C18—C17—H17B	108.5
C9—N4—H4D	122.4	H17A—C17—H17B	107.5
Cu1—N4—H4D	100.8	C17—C18—C19	106.8 (2)
H4C—N4—H4D	105.7	C17—C18—C21	111.3 (2)
C14—N5—C13	123.0 (2)	C19—C18—C21	110.2 (2)
C14—N5—H5N	113.3	C17—C18—C20	110.4 (2)
C13—N5—H5N	121.7	C19—C18—C20	110.3 (3)
C15—N6—O4	112.8 (3)	C21—C18—C20	107.8 (2)
C17—N7—H7D	120.2	C18—C19—H19A	109.5
C17—N7—H7E	95.0	C18—C19—H19B	109.5
H7D—N7—H7E	112.0	H19A—C19—H19B	109.5
C17—N7—H7F	116.4	C18—C19—H19C	109.5
H7D—N7—H7F	98.1	H19A—C19—H19C	109.5
H7E—N7—H7F	116.5	H19B—C19—H19C	109.5
C22—N8—C21	124.0 (3)	C18—C20—H20A	109.5
C22—N8—H8N	115.8	C18—C20—H20B	109.5
C21—N8—H8N	120.2	H20A—C20—H20B	109.5
C23—N9—O6	112.8 (2)	C18—C20—H20C	109.5
C25—N10—C26	124.3 (3)	H20A—C20—H20C	109.5
C25—N10—H10N	116.8	H20B—C20—H20C	109.5
C26—N10—H10N	118.2	N8—C21—C18	113.4 (2)
C31—N11—C30	124.3 (3)	N8—C21—H21A	108.9
C31—N11—H11N	116.0	C18—C21—H21A	108.9
C30—N11—H11N	119.4	N8—C21—H21B	108.9
C32—N12—O10	112.4 (2)	C18—C21—H21B	108.9
N1—C1—C2	124.0 (3)	H21A—C21—H21B	107.7
N1—C1—C3	113.6 (2)	O5—C22—N8	123.0 (3)
C2—C1—C3	122.4 (2)	O5—C22—C23	122.0 (3)
C1—C2—H2A	109.5	N8—C22—C23	115.0 (3)
C1—C2—H2B	109.5	N9—C23—C24	125.5 (3)
H2A—C2—H2B	109.5	N9—C23—C22	115.0 (2)
C1—C2—H2C	109.5	C24—C23—C22	119.5 (3)
H2A—C2—H2C	109.5	C23—C24—H24A	109.5
H2B—C2—H2C	109.5	C23—C24—H24B	109.5
O2—C3—N2	126.7 (3)	H24A—C24—H24B	109.5
O2—C3—C1	118.6 (2)	C23—C24—H24C	109.5
N2—C3—C1	114.7 (2)	H24A—C24—H24C	109.5
N2—C4—C5	113.8 (2)	H24B—C24—H24C	109.5
N2—C4—H4A	108.8	O7—C25—O8	122.7 (3)
C5—C4—H4A	108.8	O7—C25—N10	118.6 (3)
N2—C4—H4B	108.8	O8—C25—N10	118.7 (3)
C5—C4—H4B	108.8	N10—C26—C27	116.3 (3)
H4A—C4—H4B	107.7	N10—C26—H26A	108.2
C6—C5—C4	107.9 (2)	C27—C26—H26A	108.2
C6—C5—C7	110.0 (3)	N10—C26—H26B	108.2
C4—C5—C7	110.4 (2)	C27—C26—H26B	108.2
C6—C5—C8	106.5 (2)	H26A—C26—H26B	107.4
C4—C5—C8	111.3 (2)	C28—C27—C30	110.1 (3)
C7—C5—C8	110.7 (2)	C28—C27—C29	109.8 (3)
C5—C6—H6A	109.5	C30—C27—C29	107.0 (3)
C5—C6—H6B	109.5	C28—C27—C26	107.7 (3)
H6A—C6—H6B	109.5	C30—C27—C26	111.9 (2)
C5—C6—H6C	109.5	C29—C27—C26	110.3 (3)
H6A—C6—H6C	109.5	C27—C28—H28A	109.5
H6B—C6—H6C	109.5	C27—C28—H28B	109.5
C5—C7—H7A	109.5	H28A—C28—H28B	109.5
C5—C7—H7B	109.5	C27—C28—H28C	109.5
H7A—C7—H7B	109.5	H28A—C28—H28C	109.5
C5—C7—H7C	109.5	H28B—C28—H28C	109.5
H7A—C7—H7C	109.5	C27—C29—H29A	109.5
H7B—C7—H7C	109.5	C27—C29—H29B	109.5
N3—C8—C5	113.8 (2)	H29A—C29—H29B	109.5
N3—C8—H8A	108.8	C27—C29—H29C	109.5
C5—C8—H8A	108.8	H29A—C29—H29C	109.5
N3—C8—H8B	108.8	H29B—C29—H29C	109.5
C5—C8—H8B	108.8	N11—C30—C27	114.1 (2)
H8A—C8—H8B	107.7	N11—C30—H30A	108.7
N4—C9—C10	115.8 (2)	C27—C30—H30A	108.7
N4—C9—H9A	108.3	N11—C30—H30B	108.7
C10—C9—H9A	108.3	C27—C30—H30B	108.7
N4—C9—H9B	108.3	H30A—C30—H30B	107.6
C10—C9—H9B	108.3	O9—C31—N11	124.4 (3)
H9A—C9—H9B	107.4	O9—C31—C32	118.2 (3)
C11—C10—C9	110.6 (2)	N11—C31—C32	117.4 (3)
C11—C10—C13	107.2 (2)	N12—C32—C33	126.5 (3)
C9—C10—C13	112.6 (2)	N12—C32—C31	116.7 (3)
C11—C10—C12	109.7 (3)	C33—C32—C31	116.8 (3)
C9—C10—C12	107.2 (2)	C32—C33—H33A	109.5
C13—C10—C12	109.5 (2)	C32—C33—H33B	109.5
C10—C11—H11A	109.5	H33A—C33—H33B	109.5
C10—C11—H11B	109.5	C32—C33—H33C	109.5
H11A—C11—H11B	109.5	H33A—C33—H33C	109.5
C10—C11—H11C	109.5	H33B—C33—H33C	109.5
H11A—C11—H11C	109.5	N13—C35—C34	178.4 (5)
H11B—C11—H11C	109.5	C35—C34—H34A	109.5
C10—C12—H12A	109.5	C35—C34—H34B	109.5
C10—C12—H12B	109.5	H34A—C34—H34B	109.5
H12A—C12—H12B	109.5	C35—C34—H34C	109.5
C10—C12—H12C	109.5	H34A—C34—H34C	109.5
H12A—C12—H12C	109.5	H34B—C34—H34C	109.5
			
N2—Cu1—N1—C1	−5.1 (2)	C12—C10—C13—N5	−60.3 (3)
N4—Cu1—N1—C1	154.4 (2)	C13—N5—C14—O3	0.2 (4)
N2—Cu1—N1—O1	173.3 (2)	C13—N5—C14—C15	179.9 (3)
N4—Cu1—N1—O1	−27.2 (2)	O4—N6—C15—C16	−0.3 (4)
N1—Cu1—N2—C3	9.48 (19)	O4—N6—C15—C14	179.0 (2)
N3—Cu1—N2—C3	−175.8 (2)	O3—C14—C15—N6	−156.0 (3)
N4—Cu1—N2—C3	−58.1 (3)	N5—C14—C15—N6	24.2 (4)
N1—Cu1—N2—C4	176.6 (2)	O3—C14—C15—C16	23.3 (4)
N3—Cu1—N2—C4	−8.7 (2)	N5—C14—C15—C16	−156.5 (3)
N4—Cu1—N2—C4	109.0 (3)	N7—C17—C18—C19	−175.7 (2)
N2—Cu1—N3—C8	15.5 (2)	N7—C17—C18—C21	64.0 (3)
N4—Cu1—N3—C8	−144.8 (2)	N7—C17—C18—C20	−55.7 (3)
N2—Cu1—N4—C9	−18.8 (4)	C22—N8—C21—C18	−99.9 (3)
N1—Cu1—N4—C9	−85.0 (2)	C17—C18—C21—N8	59.0 (3)
N3—Cu1—N4—C9	98.9 (2)	C19—C18—C21—N8	−59.3 (3)
O1—N1—C1—C2	0.2 (4)	C20—C18—C21—N8	−179.8 (2)
Cu1—N1—C1—C2	178.6 (2)	C21—N8—C22—O5	0.5 (4)
O1—N1—C1—C3	−178.1 (2)	C21—N8—C22—C23	179.7 (2)
Cu1—N1—C1—C3	0.3 (3)	O6—N9—C23—C24	2.2 (4)
C4—N2—C3—O2	−1.0 (4)	O6—N9—C23—C22	−179.7 (2)
Cu1—N2—C3—O2	167.4 (2)	O5—C22—C23—N9	177.3 (3)
C4—N2—C3—C1	179.7 (2)	N8—C22—C23—N9	−1.9 (4)
Cu1—N2—C3—C1	−11.9 (3)	O5—C22—C23—C24	−4.5 (4)
N1—C1—C3—O2	−171.6 (2)	N8—C22—C23—C24	176.3 (3)
C2—C1—C3—O2	10.0 (4)	C26—N10—C25—O7	8.3 (4)
N1—C1—C3—N2	7.7 (3)	C26—N10—C25—O8	−174.4 (3)
C2—C1—C3—N2	−170.6 (3)	C25—N10—C26—C27	−102.4 (3)
C3—N2—C4—C5	−161.1 (2)	N10—C26—C27—C28	172.9 (3)
Cu1—N2—C4—C5	32.3 (3)	N10—C26—C27—C30	51.7 (4)
N2—C4—C5—C6	−178.5 (2)	N10—C26—C27—C29	−67.3 (3)
N2—C4—C5—C7	61.3 (3)	C31—N11—C30—C27	111.7 (3)
N2—C4—C5—C8	−62.0 (3)	C28—C27—C30—N11	−64.4 (3)
Cu1—N3—C8—C5	−47.1 (3)	C29—C27—C30—N11	176.4 (3)
C6—C5—C8—N3	−171.1 (2)	C26—C27—C30—N11	55.4 (4)
C4—C5—C8—N3	71.6 (3)	C30—N11—C31—O9	−0.2 (5)
C7—C5—C8—N3	−51.5 (3)	C30—N11—C31—C32	178.6 (3)
Cu1—N4—C9—C10	178.84 (19)	O10—N12—C32—C33	2.8 (4)
N4—C9—C10—C11	−57.4 (3)	O10—N12—C32—C31	−176.9 (2)
N4—C9—C10—C13	62.5 (3)	O9—C31—C32—N12	172.1 (3)
N4—C9—C10—C12	−177.0 (2)	N11—C31—C32—N12	−6.7 (4)
C14—N5—C13—C10	−91.1 (3)	O9—C31—C32—C33	−7.6 (4)
C11—C10—C13—N5	−179.3 (2)	N11—C31—C32—C33	173.6 (3)
C9—C10—C13—N5	58.8 (3)		

Symmetry code: (i) −*x*+2, −*y*, −*z*+1.

### Hydrogen-bond geometry (Å, º)

**Table:**

*D*—H···*A*	*D*—H	H···*A*	*D*···*A*	*D*—H···*A*
O4—H4*O*···O2^ii^	1.00	1.67	2.574 (3)	150
O6—H6*O*···O1^i^	0.91	1.71	2.613 (3)	174
N3—H3*A*···O1^i^	0.86	2.45	2.938 (3)	116
N3—H3*B*···O3	0.90	2.16	2.978 (3)	151
N4—H4*C*···O3	0.93	2.09	2.908 (3)	145
N4—H4*D*···O1	0.92	2.47	2.967 (3)	115
N4—H4*D*···N1^i^	0.92	2.50	3.123 (3)	126
N7—H7*D*···O5^iii^	0.79	2.29	3.002 (3)	149
N7—H7*D*···O5	0.79	2.60	3.109 (3)	123
N7—H7*E*···O7^iii^	0.87	1.84	2.705 (3)	172
N7—H7*F*···O8^iv^	1.05	1.82	2.859 (3)	169
N7—H7*F*···O7^iv^	1.05	2.43	2.981 (3)	112
N10—H10*N*···N13^v^	0.98	2.27	3.107 (5)	143
N11—H11*N*···O7	0.90	1.95	2.773 (3)	151
O10—H10*O*···O8^vi^	0.86	1.77	2.626 (3)	169

Symmetry codes: (i) −*x*+2, −*y*, −*z*+1; (ii) *x*, *y*+1, *z*; (iii) −*x*+2, −*y*, −*z*; (iv) *x*+1, *y*, *z*; (v) −*x*+1, −*y*+1, −*z*; (vi) −*x*+1, −*y*, −*z*.
